# Complementary Treatment with Mistletoe Extracts During Chemotherapy: Safety, Neutropenia, Fever, and Quality of Life Assessed in a Randomized Study

**DOI:** 10.1089/acm.2018.0159

**Published:** 2018-09-24

**Authors:** Florian Pelzer, Wilfried Tröger

**Affiliations:** ^1^ Society for Cancer Research, Hiscia Institute, Arlesheim, Switzerland.; ^2^ Clinical Research Dr. Tröger (CRDT), Freiburg, Germany.

**Keywords:** mistletoe therapy, chemotherapy, breast cancer, randomized clinical trial, 5-year follow-up, safety

## Abstract

***Objectives:*** Evaluate the safety and clinical response of complementary treatment with European mistletoe extracts during chemotherapy.

***Design:*** Monocentric controlled trial with 95 patients randomized into three groups.

***Settings/Location:*** National Cancer Research Center of Serbia.

***Subjects:*** Breast cancer patients (stage T_1–3_N_0–2_M_0_) undergoing surgery and adjuvant chemotherapy with six cycles of cyclophosphamide, adriamycin, and 5-fluorouracil.

***Interventions:*** Two different European mistletoe extracts (Helixor A, Iscador M Spez) were injected three times per week during 18 weeks of chemotherapy in the mistletoe group. Five-year follow-up of routine visits was documented in case report forms.

***Outcome measures:*** Safety was assessed by measuring adverse events, body temperature during chemotherapy, and probability of relapse or metastasis in a 5-year follow-up. During chemotherapy, the neutrophil count and quality of life according to EORTC QLQ-C30 were assessed.

***Results:*** The two patient groups receiving different complementary mistletoe treatments were integrated into one mistletoe group for this safety analysis. Patients in the mistletoe group did not develop more fever symptoms than patients in the control group (two short-term events in each group). No significant differences in probability of relapse or metastasis were measured between the groups (*p* = 0.7637). The mistletoe group showed a trend toward less neutropenia (*p* = 0.178) and improved pain and appetite loss scores (*p* < 0.0001 and *p* = 0.047, respectively) while having positive, but not significant, impact on other EORTC QLQ-C30 scores.

***Conclusions:*** Mistletoe extracts were safe in this clinical study. Neither did subcutaneous injections induce fever, nor did they influence the frequency of relapse and metastasis within 5 years. This result suggests that mistletoe extracts had no adverse interactions with the anticancer agents used in this study. Furthermore, certain side effects of chemotherapy decreased under this complementary treatment in breast cancer patients.

## Introduction

Extracts from the European mistletoe *Viscum album* [L.] are used in complementary and alternative medicine to treat patients in different cancer stages. In most cases, *V. album* [L.] extracts (VAEs) are injected subcutaneously during and after conventional therapies such as surgery, chemo-, hormone-, or radiotherapy. Preclinical and clinical studies have shown the safety of mistletoe extracts, and no adverse interactions with anticancer agents have been recorded yet.^1–4^ Nevertheless, more randomized clinical trials (RCTs) are required to strengthen the evidence of safety and efficacy.

VAEs have been reported to have immunomodulatory effects, among others, by increasing neutrophil counts in patients.^5,6^ However, the effect of VAEs on neutropenia occurring during chemotherapy cycles using anthracyclines still needs to be investigated in studies with sufficiently large number of patients. The immunomodulation observed under recommended VAE doses is not supposed to generate fever symptoms.^7,8^ In general, only a small, local inflammatory skin reaction at the injection site is the sign of the desired immunological reaction. While the current study has already contributed to clarify some aspects in the aforementioned field,^9–12^ this publication focuses on safety of VAEs by analyzing all measured parameters, including fever and local inflammatory skin reactions.

Most cancer patients continue VAE treatment to manage the side effects of past conventional therapies and to maintain their quality of life. In the current study, however, patients stopped the VAE treatment with the last chemotherapy cycle and were followed up for five further years. During the 18 weeks of chemotherapy, body temperature, local inflammatory skin reaction, neutropenia, and quality of life according to the European Organization for Research and Treatment of Cancer Quality of Life Questionnaire consisting of 30 questions (EORTC QLQ-C30) were assessed. In the 5-year follow-up, the disease-free survival time was measured to show whether the VAE treatment had an adverse interaction with anticancer agents.

## Methods

### Objectives

The objectives of this study were to assess the clinical response (neutropenia, fever, and quality of life according to EORTC QLQ-C30) and impact on the median disease-free survival time of VAE treatments during chemotherapy in breast cancer patients (stage T_1–3_N_0–2_M_0_).

The present publication has been designed to identify the common effects of two different VAEs given to patients in parallel with chemotherapy.

### Design

**FIG. 1.**
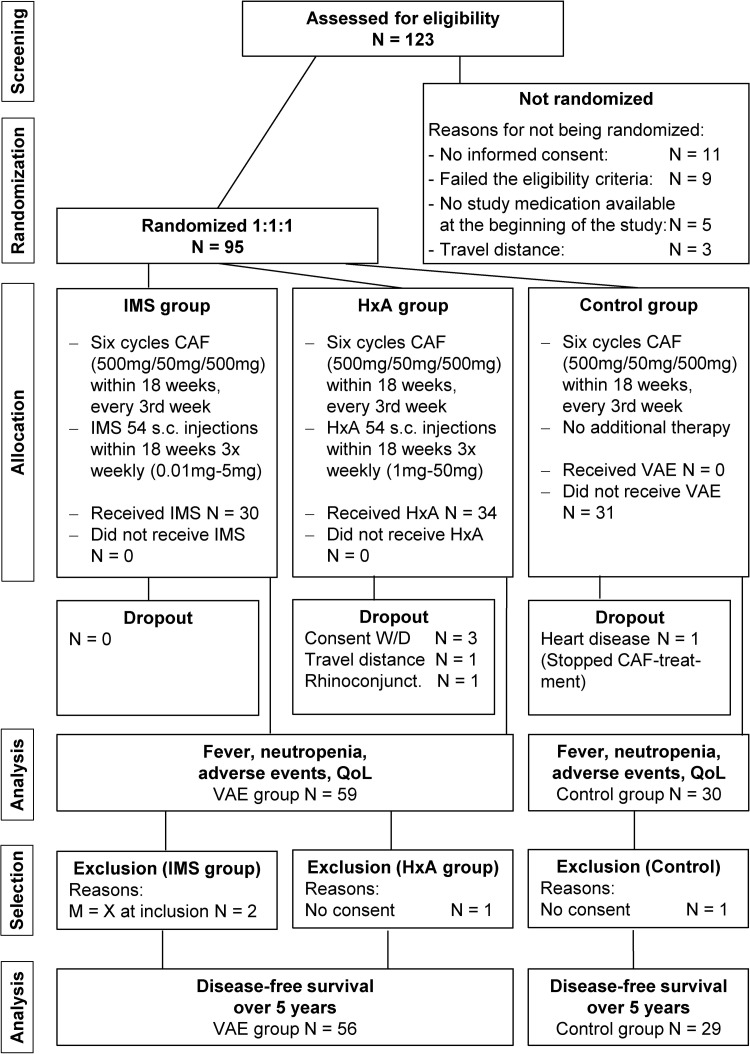
Flowchart according to CONSORT. HxA, Helixor A; IMS, Iscador® M special; VAE, *Viscum album* [L.] extracts.

A prospective, randomized, open-label clinical trial with equal size randomization into three groups (1:1:1) was conducted. Two groups received a complementary VAE treatment during the six consecutive cycles of cyclophosphamide, adriamycin, and 5-fluorouracil (CAF). These two groups were named according to the VAE administered: HxA group if Helixor A was administered and IMS group if Iscador M Spez was administered (Fig. 1). The third group received six CAF cycles without any complementary therapy and is referred to as the control group. The allocation to one group was fixed for the duration of the whole trial.

This clinical trial was conducted in two phases: the first phase lasted 18 weeks, which is the average duration of the six CAF cycles. The second phase lasted maximally 5 years, which is the duration of the follow-up.

None of the patients received VAEs after the end of chemotherapy. The present evaluation integrates the HxA and IMS groups into one group called VAE group, resulting in a *de facto* 2:1 randomization.

### Participants

Breast cancer patients in the stages T_1–3_N_0–2_ M_0_; treated at the Institute of Oncology and Radiology (IORS), National Cancer Research Center of Serbia in Belgrade; and prescribed six consecutive cycles of CAF after surgery were assessed for eligibility.

Additional inclusion criteria were female gender, age ≥18 years, Karnofsky index ≥60, leukocytes ≥3000/mm^3^, thrombocytes ≥100‚000/mm^3^, serum creatinine ≤2 mg%, serum glutamic oxaloacetic transaminase, and serum glutamic pyruvic transaminase ≤2.5 × the upper institutional limits.

Exclusion criteria were pregnancy or lactation; metastases; planned radiation or hormone therapy during six consecutive cycles of CAF; use of immunostimulant or immunosuppressive agents (e.g., corticosteroids) except for nausea and emesis; current use of other investigational agents; clinically relevant physical or mental illness such as serious infections, hepatic, renal, or other organ dysfunction or major depression; alcohol abuse, alcoholism, and oral or parenteral drug abuse; and methadone treatment.

Enrollment for this first part of the study started on December 14, 2005, and ended on February 15, 2007. In this time, 123 breast cancer patients were prescribed CAF and assessed for eligibility. Twenty-eight patients did not pass the screening, leaving 95 patients for randomization (Fig. 1).

For participation in the long-term follow-up, inclusion criteria were completion of six cycles of CAF and agreement to participate in the observation. Two patients in the VAE group were excluded due to an unknown metastatic status (M = X) before the start of chemotherapy, while one patient in the control group and one in the VAE group withdrew their consent to participate in the study. As a result, 56 of 59 patients of the VAE group and 29 of 30 patients of the control group were included in the long-term observation (Fig. 1). The follow-up began in June 2006 and ended in May 2012.

### Interventions

CAF therapy was administered to all patients in six cycles with a 3-week interval between each cycle. The scheduled dosage was 500 mg of cyclophosphamide, 50 mg of adriamycin, and 500 mg of 5 FU/1 m^2^ skin surface administered at 1 day. No other antineoplastic or immunomodulating therapies were applied during chemotherapy. All patients received antiemetic therapy with a single dose of ondansetron chloride 8 mg, dexamethasone 8 mg, and ranitidine 50 mg, respectively, administered before each CAF cycle.

Participation in the study was offered to all VAE manufacturers and therefore allowed a variety of VAEs to be included. Two manufacturers agreed to participate and decided to use mistletoes from two different host trees used in the treatment of breast cancer. Patients randomly allocated to complementary therapy with VAEs therefore received either Iscador^®^ M special (IMS) or Helixor A (HxA). IMS is a fermented aqueous extract of *V. album* [L.] from the apple tree; fresh plant material, ratio of plant to extract = 1:5, manufactured by Weleda AG, Schwäbisch Gmünd, Germany. IMS comes in 1-mL ampules for injection and each ampule contains the fermented extract of 0.01, 0.1, 1, 2, or 5 mg of fresh VAE, respectively, in isotonic saline solution. HxA is an aqueous extract of *V. album* [L.] from the fir tree; fresh plant material, ratio of plant to extract = 1:20, manufactured by Helixor Heilmittel GmbH, Rosenfeld, Germany. HxA comes in 1-mL ampules for injection, each ampule containing the aqueous extract of 1, 5, 10, 20, 30, or 50 mg of fresh mistletoe herb, respectively, in isotonic saline solution.

Both VAEs were administered by subcutaneous injections of 1 mL into the upper abdominal region three times per week (e.g., Monday, Wednesday, and Friday). The patients in the VAE groups were instructed to inject themselves subcutaneously. To investigate the safety of dosages indicated on the products' package leaflets, these same dosages were applied in this study. The dosage of VAE was increased stepwise: for IMS, 2 × 0.01, 2 × 0.1, 11 × 1, 8 × 2 mg, and remaining doses were 5 mg; for HxA, 3 × 1, 3 × 5, 3 × 10, 3 × 20, 3 × 30 mg, and the remaining doses were 50 mg. In case of local inflammatory skin reactions >5 cm or body temperature >38°C, VAE injections were reduced to the last well-tolerated dosage. An average of 53.8 ± 2.6 injections with altogether 174 mg of IMS per patient and an average of 52.3 ± 2.8 injections with altogether 2812 mg of HxA per patient were administered in the VAE group. VAE injections were stopped at the latest 3 weeks after the last CAF cycle to ensure that only direct interactions between VAEs and CAF could be the cause of different results in the 5-year follow-up.

The control group did not receive additional VAE treatment to chemotherapy.

### Outcomes

Temperature was measured sublingually in the morning on days of VAE injections. The same thermometer type (Geratherm Medical AG) was handed out to the patients for this purpose. Temperature and the diameter of local inflammatory reactions at the injection site were recorded in a diary. Unexpected rises in temperature outside the fixed measurement points were recorded in a supplementary field of the diary. Fever was defined as a temperature >38°C.

Blood samples were collected before every new CAF cycle. Neutropenia was defined as a neutrophil count of <1000/μL blood. Neutropenia and a body temperature >38°C were defined as cases of neutropenic fever.

Quality of life was documented during CAF therapy with the EORTC QLQ-C30 in the official Serbian translation.^13^ The EORTC QLQ-C30 has 30 questions summarized in 15 scores: six scores for functions and nine for symptoms. Patients filled in the EORTC QLQ-C30 seven times: once at baseline and each time before the CAF was injected.

Relapse and/or metastasis were documented annually at the prescribed routine follow-up visits to the study center for 5 years. The results were documented in case report forms designed for this study. A deviation of ±2 months was tolerated for the annual visits. The follow-up for an individual patient ended with the occurrence of a relapse or a metastasis.

### Assessment of adverse events

Adverse events were assessed by interviewing the patients and by analyzing laboratory results at each visit. Regarding local inflammatory reactions at the injection site, only those larger than 5 cm in diameter were considered as adverse events.

### Randomization

The chance to be allocated to any of the three groups (HxA group, IMS group, and control group) was 1:1:1. Variable block sizes were used for randomization without previous stratification. The randomization sequence was generated using SPSS^®^ (SPSS^®^ 14.0.1; SPSS, Inc., Chicago, IL). Allocation concealment was implemented by using sealed envelopes. Investigators at the outpatient clinic, IORS, enrolled patients, while the sealed randomization envelopes were stored in the Department of Study Coordination, IORS, and released consecutively for each enrolled patient.

### Blinding

The study was not placebo controlled as the typical and time-dependent reactions following subcutaneous VAE injections (local inflammatory reactions at the injection site, increased body temperature, and flu-like symptoms) cannot be imitated by a pseudoplacebo.

### Statistical methods

Statistical analysis was performed with SAS 9.4 and included all participating patients. The Mann–Whitney test, Fisher's exact test, Kruskal–Wallis test, and *t*-test were used to check the balance of demographic and clinical baseline characteristics and the balance of therapies after chemotherapy. The disease-free survival curves were plotted with the Kaplan–Meier method and compared using the log-rank test (Cox-Mantel).

### Adherence to regulations

The study was approved by the Ethics Committee of the National Cancer Research Center of Serbia without modifications (date: October 3, 2005) and by the Serbian Drug Agency (date: November 1, 2005). The study was conducted in compliance with the Declaration of Helsinki, Good Clinical Practice guidelines, and national laws. A registration of the study in the European Union (EU) has been rejected because Serbia is outside of the EU. All participants received patient insurance. All participants signed an informed consent before inclusion. Clinical Research Dr. Tröger (CRDT) was responsible for planning, conducting, monitoring, and analysis of the study. The two sponsors performed two audits at CRDT and one at the study site during the study. No violation of Good Clinical Practice guidelines was detected.

## Results

### Baseline data

The VAE and control groups were well balanced and did not differ significantly in their demographic and clinical characteristics (Table 1).

**Table 1.** Baseline Status

*Factor*	*VAE group*	*Control group*	p
T (TNM)	*N* = 64	*N* = 31	
1	16 (25%)	9 (30%)	
2	45 (70%)	19 (61%)	
3	2 (3%)	2 (6%)	
unk.	1 (2%)	1 (3%)	0.7519 (*χ*^2^)
N (TNM)	*N* = 64	*N* = 31	
0	26 (40%)	16 (52%)	
1	35 (55%)	14 (45%)	
2	3 (5%)	1 (3%)	0.5934 (*χ*^2^)
Grading	*N* = 64	*N* = 31	
1	1 (2%)	0 (0%)	
2	52 (80%)	24 (77%)	
3	8 (13%)	6 (20%)	
unk.	3 (5%)	1 (3%)	0.7304 (*χ*^2^)
Menopausal status	*N* = 64	*N* = 31	
Pre	30 (47%)	13 (42%)	
Peri	6 (9%)	1 (3%)	
post	27 (42%)	17 (55%)	
unk.	1 (2%)	0 (0%)	0.4954 (*χ*^2^)
Estrogen receptor status	*N* = 64	*N* = 31	
+	42 (65%)	17 (55%)	
−	19 (30%)	12 (39%)	
unk.	3 (5%)	2 (6%)	0.5958 (*χ*^2^)
Progesteron receptor status	*N* = 64	*N* = 31	
+	39 (61%)	19 (61%)	
−	22 (34%)	10 (32%)	
unk.	3 (5%)	2 (7%)	0.927 (*χ*^2^)
Age	*N* = 64	*N* = 31	
Mean ± SD	50.0 ± 7.3	50.8 ± 7.9	0.6270 (*t*-test)
BMI	*N* = 64	*N* = 31	
Mean ± SD	26.5 ± 5.3	25.6 ± 4.7	0.4185 (*t*-test)
Karnofsky	*N* = 64	*N* = 31	
Mean ± SD	100 ± 0	100 ± 0	
Pulse	*N =* 63	*N* = 31	
Mean ± SD	80 ± 13	77 ± 10	0.3177 (*t*-test)
BP systolic	*N = 64*	*N* = 29	
Mean ± SD	128 ± 17	132 ± 19	0.2945 (*t*-test)
BP diastolic	*N* = 64	*N* = 29	
Mean ± SD	81 ± 11	84 ± 14	0.3144 (*t*-test)
Temperature	*N* = 59	*N* = 30	
Mean ± SD	36.4 ± 0.4	36.5 ± 0.4	0.2406 (*t*-test)

TNM classification—T primary tumor; N lymph node metastasis; M distant metastasis; unk., unknown; Menopausal status—pre, premenopausal; peri, perimenopausal; post, postmenopausal; BMI, body mass index; BP, blood pressure; SD, standard deviation; VAE, *Viscum album* [L.] extract.

### Fever

**FIG. 2.**
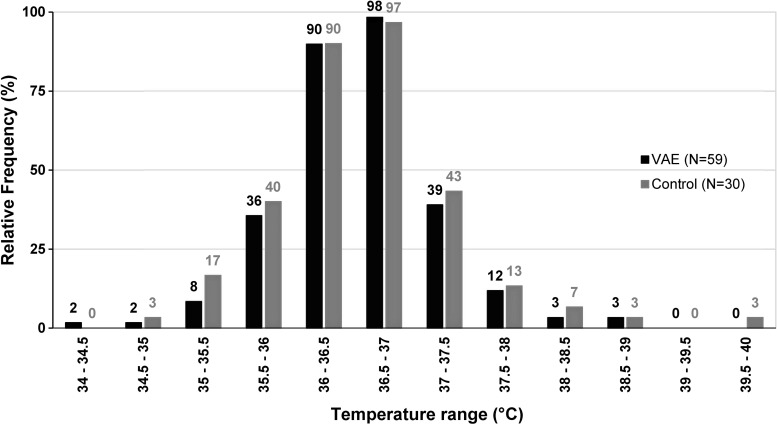
Relative frequency of recorded body temperatures. Percentages indicate the relative number of patients who recorded their body temperature at least once within the stated range. VAE, *Viscum album* [L.] extracts.

No long-lasting fever occurred in any of the two patient groups. The highest recorded temperature in the control group was 39.7°C, and the highest recorded temperature in the VAE group was 38.6°C (Fig. 2). Both patients recovered a temperature of 36.6°C and 36.2°C, respectively, at the next measurement point. In total, fever was observed in two different patients for the time span of one measurement point in both groups. VAE injections, even if causing local inflammatory skin reactions >5 cm, did not lead to an increase of the mean recorded temperature (Table 2).

**Table 2.** Temperature Data in Different Populations

	*Temperature*			
	*No. of patients*	*No. of assessments*	*Mean (°C)*	*Standard deviation*
VAE group	59	2930	36.40	0.36
Control group	30	1415	36.45	0.41
VAE patients without local skin reaction	6 (10%)	1248 (42%)	36.40	0.33
VAE patients with local skin reaction ≤5 cm	27 (46%)	1601 (55%)	36.41	0.39
VAE patients with local skin reaction >5 cm	26 (44%)	81 (3%)	36.53	0.33

### Neutropenia

Fifty-nine of 64 VAE patients and 30 of 31 control patients could be analyzed for neutropenia. Neutropenia was detected 10 times in 10 different patients (17%) of the VAE group and nine times in eight different patients (27%) of the control group (Table 3). Fisher's exact test according to the sequentially rejective Holm procedure indicates a trend (*p* = 0.178) toward less neutropenia in the VAE group. Patient #51 in the control group suffered from neutropenic fever (38.7°C), while no neutropenic fever occurred in the VAE group.

**Table 3.** List of Patients Experiencing Neutropenia During Cyclophosphamide, Adriamycin, and 5-Fluorouracil Therapy

*ID*	*Visit no.*	*Age*	*Stage*	*T*	*N*	*M*	*G*	*Neutrophils/nl*
VAE group, *N* = 59								
18	2	43	2	2	1	0	2	0.6
37	7	60	2	2	1	0	2	0.9
42	7	47	2	2	1	0	2	0.5
44	7	44	2	3	1	0	2	0.4
53	7	44	2	2	1	0	2	0.4
57	7	64	2	2	1	0	3	0.3
58	7	37	2	2	0	0	3	0.7
60	2	51	2	1	0	0	2	0.6
61	2	50	2	2	0	0	2	0.9
75	7	55	2	2	0	0	2	0.9
Control group, *N* = 30								
13	3	32	2	2	1	0	2	0.9
33	5	60	2	2	0	0	2	0.9
51	7	66	2	2	1	0	2	0.3
56	7	53	2	2	1	0	2	0.9
62	7	62	2	1	1	0	2	0.8
66	7	44	1	1	0	0	2	0.8
87	6	45	2	2	1	0	2	0.3
90	6	52	2	1	0	0	3	0.8
90	7	52	2	1	0	0	3	0.9

### Quality of life during CAF therapy

**FIG. 3.**
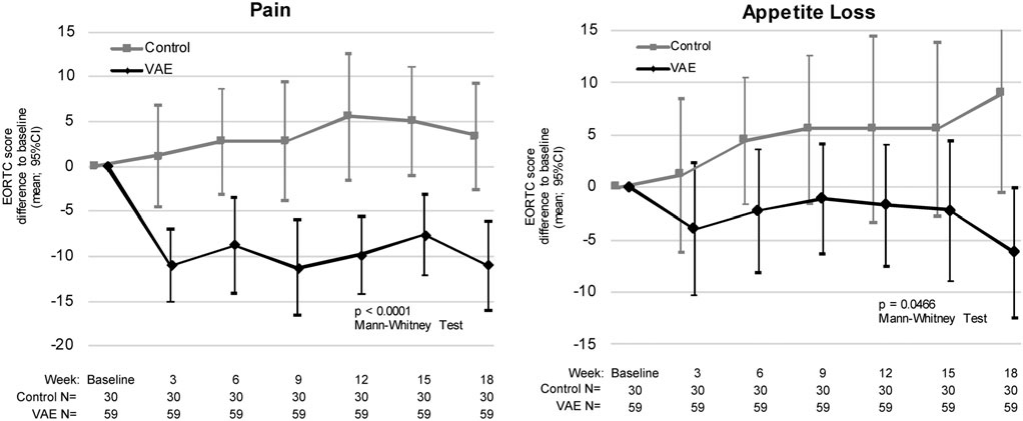
Graphs of the EORTC QLQ-C30 scores for pain and appetite loss. EORTC QLQ-C30, European Organization for Research and Treatment of Cancer Quality of Life Questionnaire consisting of 30 questions.

During chemotherapy with CAF, 3 of 15 items measured with the EORTC QLQ-C30 differed in a statistically significant and clinically relevant (score difference >7) manner between the VAE group and the control group: role functioning, pain, and appetite loss (Fig. 3 and Table 4).

**Table 4.** EORTC QLQ-C30 Scores: Mean Change from Baseline

	*Mean change from baseline*			
*EORTC QLQ-C30 item*	*VAE (*N* = 59)*	*Control (*N* = 30)*	*Mean difference*	p *(Mann–Whitney)*
Global health status	2.6	−1.9	4.4	0.2270
Physical functioning	−1.5	−4.3	2.8	0.2138
Role functioning	11.4	−2.8	14.2	<0.0001
Emotional functioning	4.0	−2.1	6.0	0.0226
Cognitive functioning	2.8	−1.6	4.4	0.2097
Social functioning	0.8	−5.9	6.7	0.0550
Fatigue	2.1	4.6	−2.5	0.2130
Nausea and vomiting	6.9	11.5	−4.6	0.1209
Pain	−9.9	3.1	−13.0	<0.0001
Dyspnea	−1.1	1.0	−2.1	0.4479
Insomnia	−2.0	6.3	−8.3	0.0675
Appetite loss	−2.9	5.2	−8.1	0.0466
Constipation	4.3	7.7	−3.3	0.5026
Diarrhea	0.0	6.3	−6.3	0.0311
Financial difficulties	8.1	10.9	−2.8	0.4868

### Adverse events

Apart from local inflammatory skin reactions at the injection site (maximal diameter observed: 12 cm), no adverse events relating to VAEs were detected.

### Disease-free survival

**FIG. 4.**
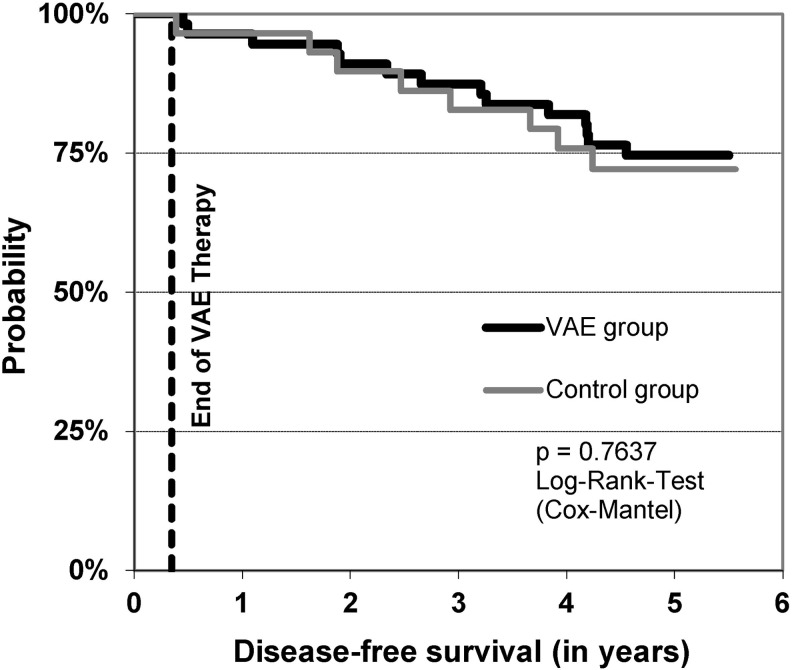
Disease-free survival of all patients.

Fifty-six of 64 VAE patients and 29 of 31 control patients could be analyzed. Fifteen of 56 patients in the VAE group and 8 of 29 patients in the control group developed relapse or metastasis within 5 years (Fig. 4). The difference between both groups is not statistically relevant (*p* = 0.7637, log-rank test). The median disease-free survival time could not be calculated because the highest probability of relapse or metastasis in 5 years was 28%.

All patients were separately analyzed according to their following adjuvant treatment, which consisted of hormone or radiotherapy. No differences could be detected in their relapse and metastasis rates (data not shown).

## Discussion

The safety of subcutaneous (s.c.) VAE injections given to cancer patients in parallel with chemotherapy has been assessed in different RCTs.^14–20^ Although the limited sample size of the present trial does not allow generalization, the results contribute to strengthen the evidence for VAE safety. A unique aspect of the present trial is the end of VAE injections with the end of chemotherapy, which allows assessing exclusively the consequences of VAE-chemotherapy interactions. *In vitro* studies showed that VAEs did not interfere with cytostatic or cytotoxic effects of a range of anticancer agents when added simultaneously to human breast, pancreas, prostate, and lung carcinoma cell lines.^3,4^ These preclinical results, together with the unaltered 5-year disease-free survival of the VAE group recorded in this study, suggest that VAEs do not have a negative impact during adjuvant chemotherapy regarding relapse and metastasis after 5 years in breast cancer patients.

Another important aspect of the present evaluation is the analysis of body temperature measurements of each patient. While past studies have recorded only single cases of fever in patients receiving s.c. VAE injections with standard dosages,^2,14,16,19,21^ one RCT has compared the body temperatures of control and VAE groups statistically without finding any difference.^21^ This is consistent with the result reported here as there is no statistical difference between the spontaneous fever events in the control group and those in the VAE group. Taken together, current data suggest that recommended doses of VAEs do not increase the probability of fever symptoms in cancer patients.

Improvement of quality-of-life parameters shown in former studies with breast cancer patients^17,18,22^ was partially corroborated in this trial; while most studies show that VAEs improve dimensions related to the patient's energy (e.g., fatigue), psychology (e.g., depression), and nutrition (e.g., appetite), there are also further studies that show a decrease in pain.^23^ The assessment of VAE effects on cancer patients' quality of life is a most promising field of research and should continue to be in the focus of future RCTs.

## Conclusion

Mistletoe extracts have been safe in this clinical study. The subcutaneous injections of mistletoe extracts neither induced fever, nor have they influenced the frequency of relapse and metastasis within 5 years. This result suggests that mistletoe extracts have had no adverse interactions with the anticancer agents in this study. Furthermore, certain side effects of chemotherapy decreased under this complementary treatment in breast cancer patients.
